# Successful Surgical Management of a Patient With Traumatic Optic Neuropathy: A Case Report

**DOI:** 10.7759/cureus.21685

**Published:** 2022-01-28

**Authors:** Dina M Abdulmannan

**Affiliations:** 1 College of Medicine, Umm Al-Qura University, Makkah, SAU

**Keywords:** surgery, surgical management, endoscopic endonasal approach, traumatic optic neuropathy, optic, neuropathy

## Abstract

A case of visual impairment following craniomaxillofacial trauma is reported. The patient had sudden visual loss associated with fracture of the left orbital floor and medial and lateral wall of the left orbit and comminuted fracture of the left optic canal. Access to the orbit was achieved through the endoscopic endonasal approach and the bone fragments, which had impinged on the optic nerve, were resected. The patient had a total return of visual acuity without surgical complications. The role of orbital and optic decompression in the management of patients with traumatic optic neuropathy is discussed. The indication of orbital and optic decompression in the management of patients with traumatic optic neuropathy is controversial and the procedure should be considered only within the context of the specific needs of the individual patient.

## Introduction

Visual impairment after facial trauma may result from injuries to the orbit and brain components responsible for vision. The most common are optic nerve or optic canal injuries. To date, no standardized treatment protocol has been developed. There are various therapeutic recommendations suggesting different treatment pathways ranging from simple clinical observation with no treatment to medical therapy with an infusion of high-dose corticosteroids to surgical decompression of the optic nerve, and finally to the combination of these two options.

Treatment of traumatic optic neuropathy could be done through the administration of high-dose steroid therapy and surgical decompression. Optimal management of traumatic optic neuropathy is controversial and depends on the nature and extent of the injury causing the visual loss. There is limited evidence on the effectiveness of steroids and surgical management concerning the benefits in the improvement of visual acuity following traumatic optic neuropathy [1,2]. The surgical approach in treating traumatic optic neuropathy is performed by decompressing the intra-canalicular optic nerve by removing structures surrounding the optic nerve or removing the bony fragments that impinge directly upon the nerve [3]. From the lateral-superior side of the optic canal, the transcranial approach can be used to decompress the optic nerve, and the transnasal approach can be used for the same from the medial-inferior part of the optic nerve [4]. The status of the optic nerve can be confirmed indirectly after traumatic optic neuropathy and there is no direct procedure to measure the pressure on the optic nerve.

Based on previous literature the treatment of traumatic optic neuropathy should be provided within 20 weeks (after trauma) as circumpapillary retinal nerve fibre layer and ganglion cell complexes no longer change after this period [4]. Additionally, it is recommended to conduct the surgery within 24 hours of steroid therapy, which should not be delayed for more than three days to have optimal outcomes concerning visual improvement [1,4,5]. The surgical approach is recommended in the following cases: failure to respond to steroid therapy, traumatic face and head injury, lack of evidence of damage or avulsion to the intracranial portion of the optic nerve, progressive loss of vision not associated with non-traumatic intraocular lesions, and the presence of bony fragments or hematoma compressing the optic nerve [4].

Findings concerning the effectiveness of surgical management of a patient with traumatic optic neuropathy are controversial. Further studies are needed to highlight patient cases for which surgical approaches could be recommended. This case report aimed to explore whether surgery could be a successful approach for the management of patients with traumatic optic neuropathy. The report presents a case of floor and medial and lateral orbit wall fractures causing visual impairment. Immediate removal of bone fragments was achieved to decompress the optic nerve.

## Case presentation

**Figure 1:**
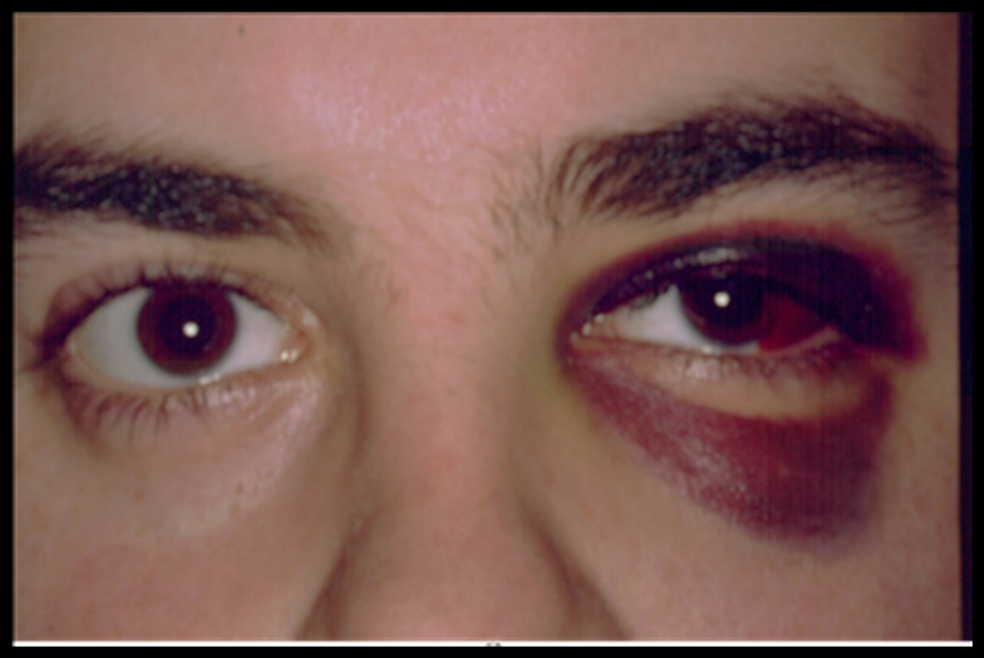
Left periorbital and eyelid ecchymosis, pupillary dilatation, and temporal sub-conjunctival haemorrhage.

A 21-year-old male sustained craniofacial trauma after a bicycle accident (no helmet). Physical examination revealed an alert, cooperative patient with left periorbital ecchymosis. Examination of the left eye revealed afferent pupillary defect and subconjunctival haemorrhage (Figure 1).

**Figure 2:**
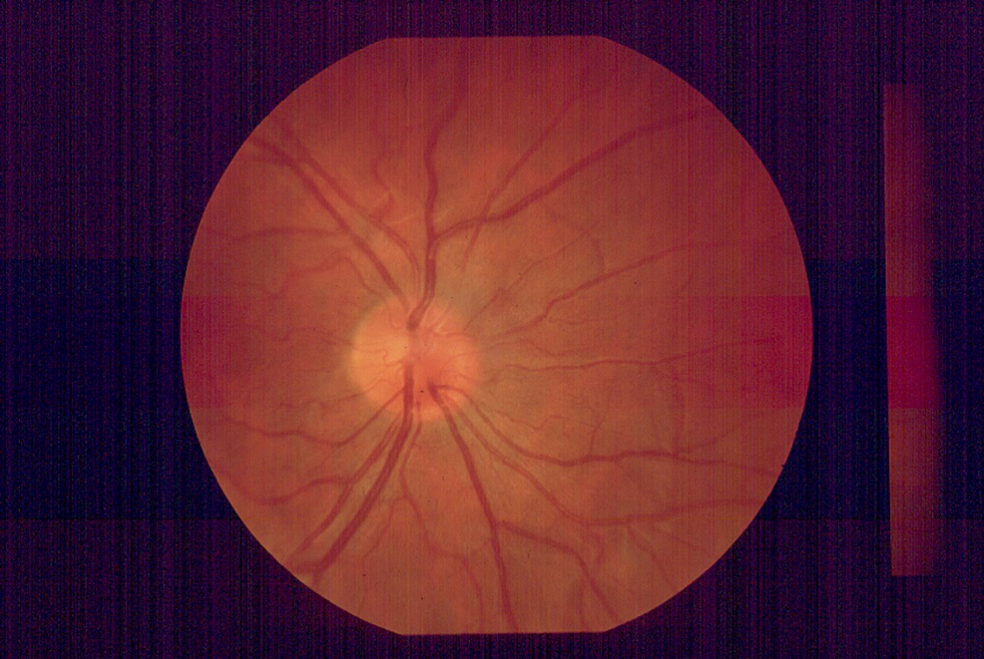
Normal appearance of optic disc in the right eye

**Figure 3:**
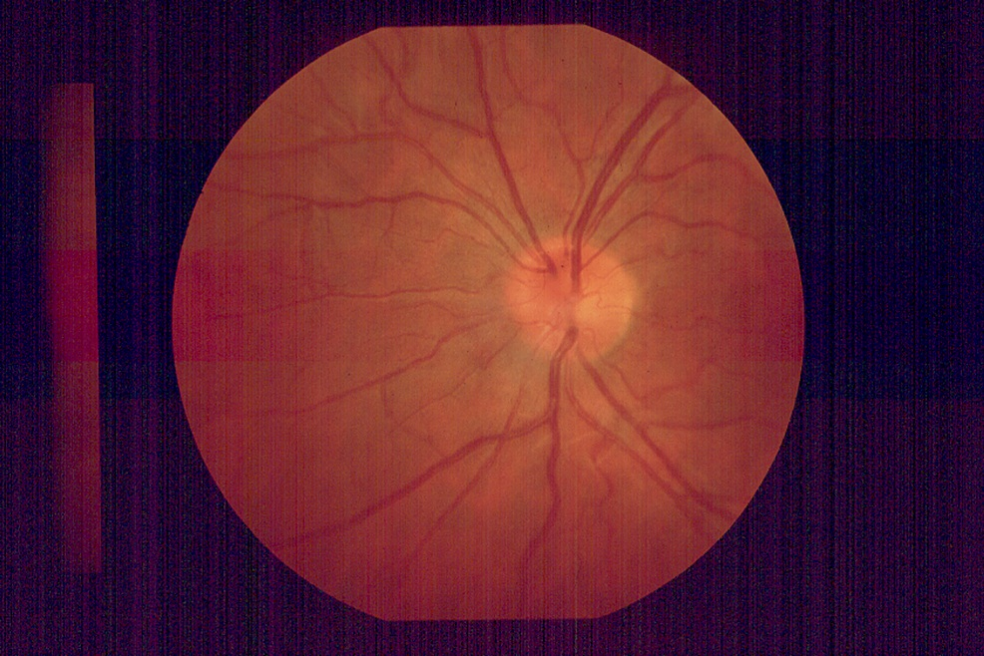
Normal appearance of optic disc in the left eye

Visual acuity was 20/20 in the right eye and, using his left eye, the patient was able to count his fingers at one meter. The eyes movement was normal before and after performing the procedure. Fundoscopic examination revealed a healthy-looking optic disc and a normally perfused retina (Figures 2-3). The intraocular pressure was 14mm in both eyes. 

**Figure 4:**
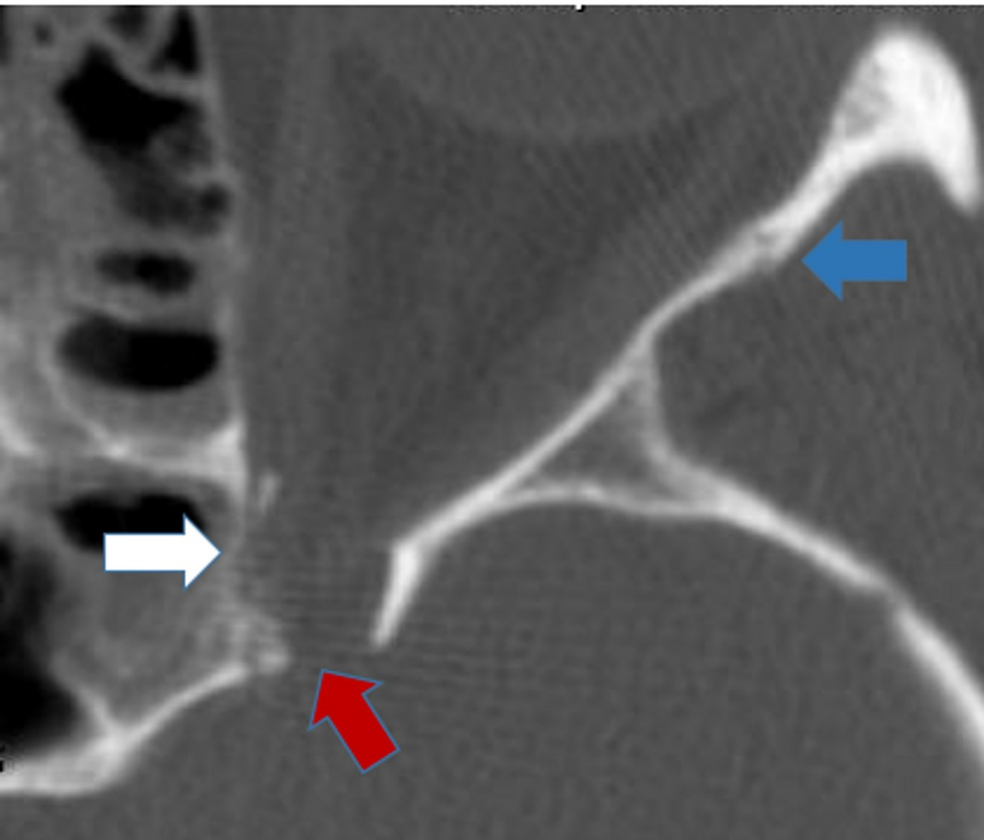
Comminuted fracture of the left optic canal Anterior clinoid process (red arrow) with optic nerve contusion along with medial (white arrow) and lateral (blue arrow) orbital walls fractures.

Radiographs revealed comminuted fractures of the left optic canal involving the anterior clinoid process and fractures of the maxillary sinus and lateral posterior wall of the sphenoid sinus (Figure 4). Two bone fragments had been displaced close to the optic nerve and were impinging on it. The major bone fragment corresponded to a portion of the lateral sphenoid bone and the minor one to a portion of the posterior sphenoidal wall. There was a fracture of the floor and medial and lateral walls of the left orbit.

Management

Intravenous methylprednisolone 1g was given for three consecutive days. After 48 hours, the patient was not improving. Therefore, the patient underwent optic nerve decompression via endoscopic endonasal approach (as it is considered the safest non-invasive approach) and the bone fragment was removed without damaging the optic nerve. Postoperatively, there was a gradual improvement in visual acuity without diplopia. The patient had 20/50 vision two weeks post-operation and 20/25 vision four weeks post operation.

## Discussion

Visual impairment in traumatic optic neuropathy occurs in 0.5-5% of closed head injuries, 45% of which are due to motor vehicle accidents, 27% to falls, 13% to assault, and 49% to bicycle accidents [6]. The optic nerve may be damaged by direct trauma resulting in penetrating injury of the optic nerve, severe orbital fracture, or penetrating orbital injury. Another mechanism of injury is compression, where a bone fragment and fracture of the optic canal or haematoma within the optic canal can lead to damage to the optic nerve, which is characterised by immediate vision loss [7-9]. Ophthalmoscopy shows a complete or partial absence of the optic disc and pre-papillary haemorrhage, and there is no known treatment available.

Indirect trauma is the most common form of traumatic optic neuropathy caused by blunt head trauma that produces acceleration-deceleration injury [8,10]. In a frontal head injury, the force is transmitted posteriorly through the orbital walls to the sphenoid bone optic canal. Most optic nerve lesions are believed to be related to damage to the intracanalicular segment of the nerve, but displaced bone fragments anywhere in the orbital stretch will damage it. Clinical assessment of the pupillary size and reactivity to light is essential. The presence of Marcus Gunn pupil with a normal fundoscopic examination is pathognomonic for afferent optic nerve injury.

Computed tomography (CT) is essential to delineate the nature and location of the injury and allow optimal views of the optic nerve, optic canal, and the intracranial lesion that is disturbing visual acuity. Numerous therapeutic protocols exist for traumatic optic neuropathy because no single treatment has proved optimal. The treatment occurs either by observation, administration of steroids, or surgical decompression [11]. According to the International Optic Nerve Trauma Study (IONTS), treatment with a megadose of corticosteroids stabilises the lipid membrane, increases blood supply, reduces tissue oedema and thus suppresses chemical cascade, which leads to cord injury [11]. A National Acute Spinal Cord Injury Study showed that no clear benefit was found for either corticosteroid therapy or optic canal decompression surgery. Additionally, there was no indication that the dosage or timing of corticosteroid treatment or the timing of surgery was associated with an increased probability of visual improvement [10]. On the other hand, other studies that explored the effect of steroids therapy in combination with optic nerve decompression surgery in traumatic optic neuropathy reported that steroids had no additional beneficial effect on the visual outcome for patients who were treated with optic nerve decompression surgery [11,12]. Treatment with optic canal decompression relieves intra-canalicular pressure and annular strangulation; removal of impinging bone fragments, and drainage of intra-canalicular hematoma must also take place [4].

There has been controversy about the surgical indication for a long time, and in early reports, surgery was undertaken only if there was an obvious fracture of the optic canal because craniotomy or trans-facial approaches had a high rate of complications [1]. The rationale for surgical decompression is that oedema of the optic nerve or haemorrhage within the bony canal impairs the nerve's blood supply, resulting in ischemia and additional damage to the injured optic nerve. The rationale behind optic nerve decompression is to partially remove the optic canal in order to decompress the nerve within the canal, limiting the detrimental effects of compression and restoring nerve function. Decompression of the optic nerve lowers intracanalicular pressure and enables the removal of any impinging bone fragments, allowing nerve function to be restored. Systematic steroids have a similar effect, leading to ‘‘medical decompression’’ [13]. There are numerous surgical approaches for optic nerve decompression that have been proposed (transfrontal craniotomy, orbitotomy, and transethmoidal and sphenoethmoidal surgery). The method chosen must be carefully evaluated in light of the location and extent of the orbital injury to select the most direct approach with the least morbidity [4]. A previous study reported the effectiveness of multiple procedures (endoscopic endonasal, transcranial, transethmoidal, and endoscopic assisted medial transorbital) concerning traumatic optic neuropathy surgery and concluded that visual improvement after surgical procedure ranged between 40-75% if it was performed in the early stage [4]. The endonasal approach is less invasive and shows excellent exposure to the optic canal and orbital apex than the classic transcranial approach. Endoscopic optic nerve surgery is better tolerated by compromised patients, has a shorter operative time, preserves olfaction, avoids external scars, and reduces morbidity. Patients of a younger age group have a better prognosis for visual acuity outcomes [13,14].

Future large-scale studies are recommended to explore the effectiveness of this approach on patients with different types of trauma and different degree of severity.

## Conclusions

Surgical management of patients with traumatic optic neuropathy is a promising approach. Patients and their families should be informed of the diagnosis and its implications. They should be aware that, although medical and surgical treatment is available, no validated approach to its management exists as yet, as the current evidence concludes that neither corticosteroids nor optic canal surgery should be considered the standard of care for patients with traumatic optic neuropathy. It is therefore clinically reasonable to decide the management of individual patients on a case-by-case basis.
